# Deep sequencing of the *Camellia sinensis *transcriptome revealed candidate genes for major metabolic pathways of tea-specific compounds

**DOI:** 10.1186/1471-2164-12-131

**Published:** 2011-02-28

**Authors:** Cheng-Ying Shi, Hua Yang, Chao-Ling Wei, Oliver Yu, Zheng-Zhu Zhang, Chang-Jun Jiang, Jun Sun, Ye-Yun Li, Qi Chen, Tao Xia, Xiao-Chun Wan

**Affiliations:** 1 Key laboratory of Tea Biochemistry and Biotechnology, Ministry of Education, Ministry of Agriculture, Anhui Agricultural University, Hefei, 230036, PR China; 2 Donald Danforth Plant Science Center, 975 North Warson Road, Saint Louis, Missouri, 63132, USA

## Abstract

**Background:**

Tea is one of the most popular non-alcoholic beverages worldwide. However, the tea plant, *Camellia sinensis*, is difficult to culture *in vitro*, to transform, and has a large genome, rendering little genomic information available. Recent advances in large-scale RNA sequencing (RNA-seq) provide a fast, cost-effective, and reliable approach to generate large expression datasets for functional genomic analysis, which is especially suitable for non-model species with un-sequenced genomes.

**Results:**

Using high-throughput Illumina RNA-seq, the transcriptome from poly (A)^+ ^RNA of *C. sinensis *was analyzed at an unprecedented depth (2.59 gigabase pairs). Approximate 34.5 million reads were obtained, trimmed, and assembled into 127,094 unigenes, with an average length of 355 bp and an N50 of 506 bp, which consisted of 788 contig clusters and 126,306 singletons. This number of unigenes was 10-fold higher than existing *C. sinensis *sequences deposited in GenBank (as of August 2010). Sequence similarity analyses against six public databases (Uniprot, NR and COGs at NCBI, Pfam, InterPro and KEGG) found 55,088 unigenes that could be annotated with gene descriptions, conserved protein domains, or gene ontology terms. Some of the unigenes were assigned to putative metabolic pathways. Targeted searches using these annotations identified the majority of genes associated with several primary metabolic pathways and natural product pathways that are important to tea quality, such as flavonoid, theanine and caffeine biosynthesis pathways. Novel candidate genes of these secondary pathways were discovered. Comparisons with four previously prepared cDNA libraries revealed that this transcriptome dataset has both a high degree of consistency with previous EST data and an approximate 20 times increase in coverage. Thirteen unigenes related to theanine and flavonoid synthesis were validated. Their expression patterns in different organs of the tea plant were analyzed by RT-PCR and quantitative real time PCR (qRT-PCR).

**Conclusions:**

An extensive transcriptome dataset has been obtained from the deep sequencing of tea plant. The coverage of the transcriptome is comprehensive enough to discover all known genes of several major metabolic pathways. This transcriptome dataset can serve as an important public information platform for gene expression, genomics, and functional genomic studies in *C. sinensis*.

## Background

The tea beverage yields many health benefits to humans due to the extensive secondary metabolites in tea leaves, including polyphenols, theanine, and volatile oils [1,2]. The tea plant, *Camellia sinensis*, is both an economically important horticultural crop and a model system for studying self-incompatibility and Theaceae plants [3]. Due to its large genome (4.0 Gigabases) [4] and lacks of developed genetic tools such as tissue culture and transformation, little genomic information is available. As of August 2010, only 810 nucleotide sequences, 12,664 expressed sequence tags (ESTs), 1 genome survey sequence (GSS), and 478 proteins from *C. sinensis *have been deposited in GenBank. The bulk of tea research has focused on analysis of secondary metabolism genes, which were mostly discovered through EST sequencing. In one study, 588 ESTs derived from a subtractive cDNA library were sequenced. Approximately 8.7% of these ESTs were related to secondary metabolism, including leucoanthocyanidin reductase (LCR) involved in catechin synthesis [5]. In another study, 4.8% of the 1,684 ESTs in a tender shoot cDNA library were related to secondary metabolism, including chalcone isomerase (CHI) [6]. Moreover, in a young tea root cDNA library constructed in our laboratory, 4.5% of the 4,860 valid ESTs were secondary metabolic genes [7].

EST sequencing has long been the core technology for reference transcript discovery [8-10]. However, EST sequencing has some inherent limitations, such as low throughput, high cost, and lack of quantitation of the expressed genes. In addition, there is some bias in the composition of cDNA libraries caused by bacterial cloning, such as vector contamination, overrepresentation of preferentially cloned sequences, and inadequate representation of rare or inherently unclonable transcripts [11-13]. These inherent limitations of EST sequencing and the small number of available ESTs suggested that our understanding of the tea transcriptome is far from complete.

The cost-effective and ultra-high-throughput DNA sequencing technology, RNA-seq, is a revolutionary advance in genome-scale sequencing. This transcriptome analysis method is fast and simple because it does not require bacterial cloning of the cDNAs. Direct sequencing of these cDNAs can generate short reads at an extraordinary depth. Following sequencing, the resulting reads can be assembled into a genome-scale transcription profile. It is a more comprehensive and efficient way to measure transcriptome composition, obtain RNA expression patterns, and discover new exons and genes [11-16]. Recent transcriptomic studies on yeast, *Arabidopsis thaliana*, mouse, and human cells have demonstrated that this approach is well-suited for surveying the complexity of transcription in eukaryotes [11,17-23]. Since RNA-seq is not limited to detecting transcripts that correspond to existing genomic sequences, it is particularly attractive for non-model organisms with genomic sequences that are yet to be determined [24-27]. In addition, this new approach is very sensitive, allowing detections of low abundant transcripts.

In this report, we took advantage of RNA-seq to survey the poly (A)^+ ^transcriptome of *C. sinensis*. The coverage of the transcriptome, at 2.59 gigabase pairs, was comprehensive enough to discover all known genes of several major metabolic pathways. This transcriptome dataset will serve as a public information platform for gene expression, genomics, and functional genomics in *C. sinensis*.

## Results

### Sequencing, *de novo *assembly, and sequence analysis

**Figure 1:**
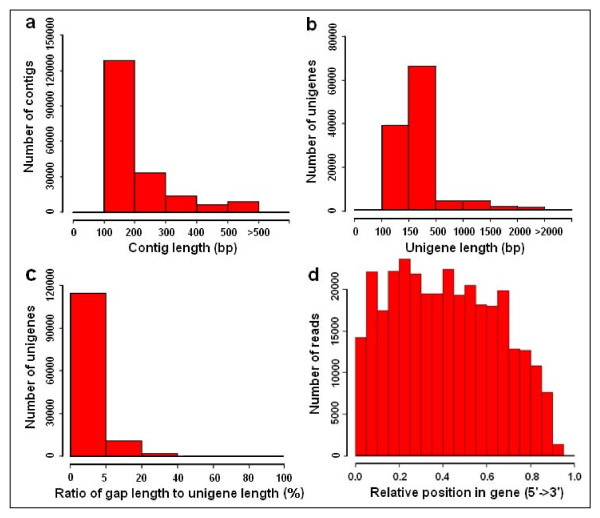
**Overview of the *C. sinensis *transcriptome assembly**. (a) Size distribution of the contigs obtained from *do novo *assembly of high-quality clean reads. (b) Size distribution of the unigenes produced from further assembly of contigs by contig joining, gap filling, and scaffold clustering. (c) Ratio distribution of the gap's length to the length of assembled unigenes. The x-axis indicates the ratio of the gap's length to the length of assembled unigenes. The y-axis indicates the number of unigenes containing gaps. (d) Random distribution of Illumina sequencing reads in the assembled unigenes. The x-axis indicates the relative position of sequencing reads in the assembled unigenes. The orientation of unigene is from 5' end to 3' end.

To comprehensively cover the tea transcriptome, total RNA was extracted from seven different tissues: tender shoots, young leaves, mature leaves, stems, young roots, flower buds and immature seeds. Equal amounts of total RNA from each tissue were pooled together. The poly (A)^+ ^RNA was isolated, sheered into smaller fragments, and reverse-transcribed to synthesize cDNA for Illumina GA IIx sequencing. A total of 34.5 million reads 75 bp long (including single-end reads and paired-end reads) were obtained from one plate (8 lanes) in a single sequencing run, generating approximate 2.59 gigabase pairs (Gbp) of raw data. An overview of the sequencing and assembly is outlined in Table 1. After removal of adaptor sequences, duplication sequences, ambiguous reads and low-quality reads, 30.9 million high-quality clean reads (2.32 Gbp, 89.6% of the raw data) remained. The quality of the clean reads data was assessed based on the base-calling quality scores from the Illumina's base-caller Bustard. Eighty-one percent of the clean reads data (1.88 Gbp, data not shown) has Phred-like quality scores at the Q20 level (an error probability of 0.01). All high-quality reads were assembled *de novo *by SOAPdenovo program [28], producing 191,376 contigs longer than 100 bp (amounting to 39.72 Mbp), with an average contig length of 208 bp and an N50 of 225 bp (i.e. 50% of the assembled bases were incorporated into contigs 225 bp or longer). The size distribution of these contigs is shown in Figure 1a. Although the majority of the contigs are between 100 to 300 bp, we obtained 9,209 contigs which were greater than 500 bp in length.

**Table 1:** Summary of sequence assembly after Illumina sequencing

	Sequences (n)	Base pairs (Mbp)	Mean length (bp)	N50 (bp)
Raw sequencing reads	34,493,882	2,587.04	75	-
Clean reads	30,929,074	2,319.68	75	-
Contigs (≥100 bp)	191,376	39.72	208	225
Scaffold sequences (≥100 bp)	127,901	45.07	355	506
Singletons (≥100 bp)	126,306	-	-	-
Clusters (≥100 bp)	788	-	-	-
Total unigenes (≥100 bp)	127,094	45.07	355	506

The data from the paired-end sequencing was used to join the contigs into scaffolds, which represent the collections of fragments originating from a single transcript. Overall, the contig-joining procedure based on paired-end reads condensed the number of contigs from 191,376 to 127,901. Among them, 119,105 contigs (62.2% of total) merged into 55,630 unique scaffolds.

Subsequently, gap filling and contig joining were carried out to complete as many scaffolds as possible. Through scaffold assembly, an additional 5.35 Mbp were incorporated into existing scaffolds. The amount of all processed data totalled 45.07 Mbp, which further improved the quality of *de novo *assembly of these short reads.

Assembly of transcriptome sequencing reads can produce more scaffolds than expressed genes, reflecting redundancy among the assembled sequences (i.e., more than one sequence per gene). To reduce any redundancy, assembled sequences from the above analysis were clustered using the Gene Indices Clustering Tools [29]. Sequence clustering merged 1,596 scaffolds into 788 sequence clusters (also known as "consensuses"). These sequence clusters had 2-4 scaffolds per cluster, and 99% of the clusters contained two scaffolds. The remaining 126,306 scaffolds were retained as singletons, for a total of 127,094 "unigenes". This result demonstrated that the assembly and contig joining succeeded in processing a large amount of short reads from the tea plant samples with relatively little redundancy.

Out of the 127,094 unigenes, 22,757 unigenes were ≥500 bp and 7,820 were ≥1,000 bp, with an average unigenes length of 355 bp and an N50 of 506 bp. The size distribution for these unigenes is shown in Figure 1b. The unigene distribution followed the contig distribution closely, with the majority being shorter sequences.

To evaluate the quality of the dataset, we analyzed the ratio of the gap's length to the length of assembled unigenes (Figure 1c). The majority of the unigenes showed gap lengths that were less than 5% of the total length, which accounted for 90.0% of total unigenes numbers (114,346 unigenes). In addition, sequencing bias was analyzed by detecting random distribution of reads in assembled unigenes (Figure 1d). Although the 3' ends of all assembled unigenes contained relatively fewer numbers of reads, other positions of all assembled unigenes showed greater and more even distribution. This observation is consistent with previous publications [27], suggesting that the quality of our dataset was comparable to similar reports in other non-model species.

### Functional annotation and classification of the *C. sinensis *transcriptome

Annotation of the *C. sinensis *transcriptome sequences was based on two levels of sequence similarity, namely sequence-based and domain-based alignments. Sequence-based alignments were performed against three public databases, including the non-redundant protein database (NR) at NCBI, Swiss-Prot/Uniprot, and the Kyoto Encyclopedia of Genes and Genomes database (KEGG) using BLASTX algorithm with a significant E-value threshold of 1e^-5^. Domain/family searches contained Hidden Markov Model (HMM) domain/family searches in both the InterPro and Pfam databases and BLASTX alignments against the Clusters of Orthologous Groups (COGs) database at NCBI. The E-value thresholds were also set at ≤1e^-5^. Annotations of the best BLASTX hits and domain hits are summarized in Table 2.

**Table 2:** Summary of annotations of the *C. sinensis *unigenes

	Sequences (n)	Annotations (n)	Functional classification
All assembled unigenes	127,094	-	-
Gene annotations against plant proteins of NR	41,483	41,483	-
Gene annotations against *Arabidopsis *protein of NR	50,214	50,214	
Unique gene annotations against NR	53,937	53,937	
Gene annotations against UniProt	25,462	25,462	-
Gene annotations against InterPro	32,646	32,646	3,485 domains/families
Gene annotations against Pfam	40,638	40,638	6,673 domains/families
Gene annotations against COG	11,241	15,701	24 categories
Gene annotations against KEGG	16,939	16,939	214 pathways
GO annotations for NR protein hits	3,577	14,283	3 main categories 43 sub-categories
GO annotations for *Arabidopsis *protein hits	32,017	157,650	3 main categories 41 sub-categories
All annotated Unigenes	55,088	-	-
Unigenes matching all six databases	9,139	-	-

#### Annotation of predicted proteins

**Figure 2:**
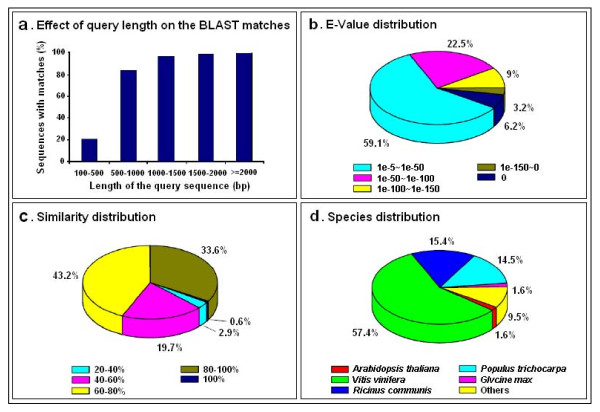
**Characteristics of homology search of unigenes against the NR database**. (a) Effects of query sequence length on percentage of significant matches. The cut-off value was set at 1.0e^-5^. The proportion of sequences with matches in the NR database at NCBI is greater among the longer assembled sequences. (b) E-value distribution of the top BLAST hits for each unigene (E-value of 1.0e^-5^). (c) Similarity distribution of the best BLAST hits for each unigene. (d) Species distribution is shown as the percentage of the total homologous sequences (with an E-value ≤ 1.0e^-5^). We used all plant proteins in the NCBI NR database for homology search and extracted the best hit of each sequence for analysis.

For annotations of gene names, coding sequences (CDS), and predicted proteins, all assembled sequences were first searched against plant proteins of the NR database, which returned 41,483 significant BLAST hits (32.6% of all unigenes; see Table 2 and Additional File 1). The length of query sequences was crucial in determining the level of significance of the BLAST match. As shown in Figure 2a, only 20.5% of the unigenes shorter than 500 bp could achieve significant BLAST scores in the NR database. In contrast, the proportion of unigenes with significant BLAST scores increased sharply to: 83.3% for query sequences between 500 and 1,000 bp; 96.5% for query sequences between 1,000 and 1,500 bp; 98.6% for query sequences between 1,500 and 2,000 bp; and 99.9% for query sequences ≥2,000 bp. The result indicates that the proportion of sequences with matches in the NR database is greater among the longer assembled sequences. Similar analytical results were obtained using the other five databases (data not shown). The E-value distribution of the top hits in the NR database showed that 40.9% of the mapped sequences have strong homology (smaller than 1.0e^-50^), while the other 59.1% of the homologous sequences ranged between 1.0e^-5 ^to 1.0e^-50 ^(Figure 2b). The similarity distribution showed 34.2% of the query sequences have a similarity higher than 80%, while 65.8% of the hits have a similarity ranging from 20% to 80% (Figure 2c). Homologous genes come from several species, with 57.4% of the unigenes have the highest homology to genes from *Vitis vinifera*, followed by *Ricinus communis *(15.4%), *Populus trichocarpa *(14.5%), *Arabidopsis thaliana *(1.6%) and *Glycine max *(1.6%) (Figure 2d).

**Figure 3:**
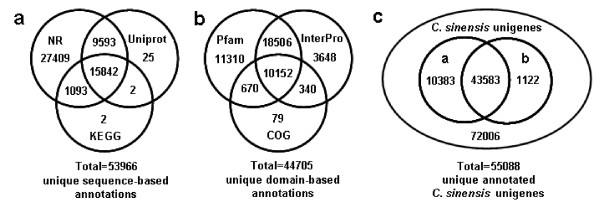
**Venn diagram showing distribution of similarity search results**. (a) The number of unique sequence-based annotations is the sum of unique best BLASTX hits from the NR (NR counts including the unique BLASTX hits from the plant proteins and *Arabidopsis *proteins), Uniprot and KEGG databases (E-value ≤ 1.0e^-5^), respectively. The overlap regions among the three circles contain the number of unigenes that share BLASTX similarity with respective databases. (b) The number of unique domain-based annotations is the integration of unique similarity search results against the InterPro, Pfam and COGs databases (E-value ≤ 1.0e^-5^), respectively. (c) Number of all annotated *C. sinensis *unigenes is figured out based on the summation of both unique sequence-based annotations and unique domain-based annotations. The circle "a" and "b" indicate the two subsets of *C. sinensis *unigenes with sequence-based annotations (53,966 counts in Figure 3a) and domain-based annotations (44,705 counts in Figure 3b), respectively.

Because of the relatively short length of the unigenes (mean size of 355 bp) and the lack of *C. sinensis *genome information, two-thirds of the unigenes did not have a match in the plant protein dataset of NR database. Furthermore, 77 percent of the above mentioned 41,483 annotation descriptions were uninformative (e.g., 'unknown', 'unnamed', 'putative', or 'hypothetical' protein). The protein dataset of the model dicot plant *Arabidopsis *(from NR) and Swiss-Prot/Uniprot database, both of which contain highly annotated protein sequences, were used for additional BLAST alignments to produce more definitive annotations. As a result, there were 50,214 and 25,462 high-score BLAST matches from the comparisons with *Arabidopsis *and Uniprot proteins, respectively (Table 2). Most importantly, 89.9% (45,126) of *Arabidopsis *hits and 85.3% (21,720) of Uniprot hits matched genes with known functions (data not shown). By integration of similarity search results from plant proteins and *Arabidopsis *proteins, a total of 53,937 unique best BLASTX matches were produced from NR (Table 2), and the unigenes obtaining gene descriptions from NR or Uniprot amounted to 53,964 (shown in Figure 3a). These detailed annotations were valuable in the assignments of putative functions (gene names and 'CDS' or protein sequences) to the unigenes.

#### Conserved domain annotation and COG classification

Considering that different genes can share certain protein domains, reflecting a level of sequence similarity not accounted for by simple sequence-based alignments, conserved domains in *C. sinensis *unigenes were further identified against the InterPro, Pfam and COGs databases. Searches against the InterPro database [30] revealed 32,646 top hits categorized into 3,485 domains/families (Table 2 and Figure 3b). Most domains were found containing 1-3 sequences, with a small proportion appearing more frequently. InterPro domains/families were ranked according to the number of *C. sinensis *unigenes contained in each InterPro domain, and the 30 most abundant InterPro domains/families are represented in Table 3. Among these protein domains/families, "Protein kinase" and its subcategories "Serine/threonine-protein kinase" and "Tyrosine-protein kinase", known to regulate the majority of cellular pathways, were ranked in the top. Moreover, highly represented were "Cytochrome P450" and "UDP-glucosyltransferase" families, which might contribute to extensive modifications of various secondary compounds, the "WD40-repeat" domain, associated with regulation of signal transduction, transcription and proliferation [31], and the "Heat shock protein" family, related to defense mechanisms of thermal stress. In addition, 40,638 of the assembled unigenes matched entries in the Pfam database [32], corresponding to 6,673 different domains/families (Table 2 and Figure 3b). The top 8 most frequently detected domains contained "Protein kinase," "Tyrosine-protein kinase," "Pentatricopeptide repeat," "Cytochrome P450," "UDP-glucosyltransferase," "RNA recognition motif," "WD40-repeat," and "Reverse transcriptase (RNA-dependent DNA polymerase) " (see Additional File 2). The top 8 Pfam results were all in the above mentioned 30-most-abundant InterPro domain list.

**Table 3:** The 30 most frequently occurring InterPro domains/families in *C. sinensis *unigenes

Conserved domain/family	Accession ID	Sequences (n)	Rank
Protein kinase, catalytic domain	IPR000719	1415	1
Serine/threonine-protein kinase-like domain	IPR017442	1017	2
Pentatricopeptide repeat	IPR002885	636	3
Serine/threonine-protein kinase domain	IPR002290	549	4
Leucine-rich repeat	IPR001611	475	5
Tyrosine-protein kinase, subgroup, catalytic domain	IPR020635	450	6
Cytochrome P450	IPR001128	366	7
Tyrosine-protein kinase, catalytic domain	IPR001245	308	8
WD40-repeat-containing domain	IPR017986	284	9
Zinc finger, RING-type	IPR001841	278	10
WD40 repeat, subgroup	IPR019781	271	11
WD40 repeat 2	IPR019782	260	12
UDP-glucuronosyl/UDP-glucosyltransferase	IPR002213	258	13
RNA recognition motif, RNP-1	IPR000504	253	14
WD40 repeat	IPR001680	248	15
RNA-directed DNA polymerase (reverse transcriptase), related	IPR015706	243	16
Zinc finger, C3HC4 RING-type	IPR018957	237	17
ATPase, P-type, K/Mg/Cd/Cu/Zn/Na/Ca/Na/H-transporter	IPR001757	228	18
Molecular chaperone, heat shock protein, Hsp40, DnaJ	IPR015609	181	19
Myb-type HTH DNA-binding domain	IPR017930	169	20
Cyclin-like F-box	IPR001810	164	21
Myb, DNA-binding	IPR014778	163	22
Ankyrin repeat-containing domain	IPR020683	153	23
EF-HAND 2	IPR018249	152	24
Leucine-rich repeat, N-terminal	IPR013210	151	25
Tetratricopeptide region	IPR013026	145	26
Protein phosphatase 2C	IPR015655	145	26
NB-ARC	IPR002182	143	27
Myb transcription factor	IPR015495	130	28
Peptidase S8, subtilisin-related	IPR015500	130	28
Ankyrin repeat	IPR002110	130	28
Mitochondrial substrate carrier	IPR001993	130	28
ATPase, AAA-type, core	IPR003959	128	29
Mitochondrial substrate/solute carrier	IPR018108	122	30

**Figure 4:**
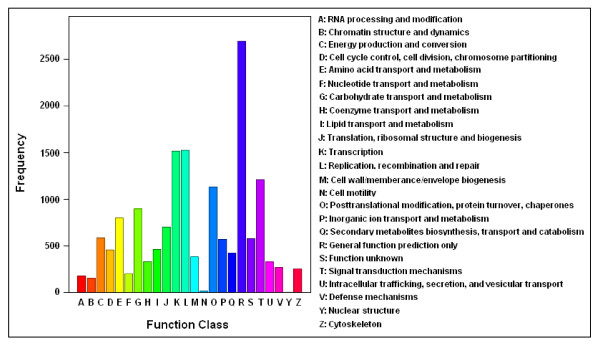
**COG Function Classification of the *C. sinensis *transcriptome**. A total of 11,241 unigenes showing significant homology to the COGs database at NCBI (E-value ≤ 1.0e^-5^) have a COG classification among the 24 categories.

More specifically, the assembled unigenes were compared against COGs [33] for in-depth analysis of phylogenetically widespread domain families. COGs consist of protein sequences encoded in 21 complete genomes, including bacteria, algae and eukaryotes, and were built on classifications according to phylogenetic relationships. Each COG consists of individual proteins or groups of paralogs from at least three lineages and thus corresponds to an ancient conserved domain. From *C. sinensis *unigenes set, 11,241 of the assembled sequences showing significant homology were assigned to the appropriate COG clusters (Table 2 and Figure 3b). Because some of these unigenes were annotated with multiple COG functions, altogether 15,701 functional annotations were produced. These COG classifications were grouped into 24 function categories (Figure 4). The five largest categories include 1) "general functions" (17.1%) associated with basic physiological and metabolic functions; 2) "replication, recombination and repair" (9.7%); 3) "transcription" (9.7%); 4) "signal transduction mechanisms" (7.7%); and 5) "posttranslational modification, protein turnover and chaperones" (7.2%). The category of secondary metabolism was highlighted with 2.7% (427 of the functional genes), because of the importance of secondary metabolites to the quality and taste of tea. The most abundant sequences in this category are cytochrome P450 monooxygenases, with a total of 147 unigenes involved in various metabolic pathways. In total, 44,705 unique domain-based annotations from InterPro, Pfam and COGs were assigned to *C. sinensis *unigenes (Figure 3b).

#### Gene Ontology (GO) Classification

**Figure 5:**
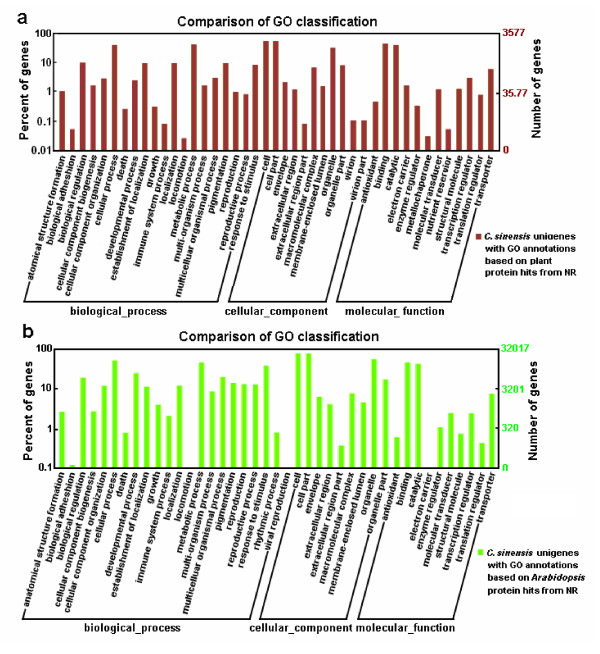
**Gene Ontology Classification of the *C. sinensis *transcriptome**. (a) Gene ontology (GO) term assignments to *C. sinensis *unigenes based on significant plant species hits against the NR database are summarized into three main GO categories (biological process, cellular component, molecular function) and 43 sub-categories. (b) Gene ontology (GO) term assignments to *C. sinensis *unigenes based on high-score BLASTX matches to the *Arabidopsis *proteins of NR database are classified into three main GO categories and 41 sub-categories. The left y-axis indicates the percentage of a specific category of genes in that main category. The right y-axis indicates the number of genes in the same category.

To functionally categorize *C. sinensis *unigenes, Gene Ontology (GO) terms were assigned to each assembled unigene. GO terms are dynamically-structured control vocabulary that can be applied to describe functions of genes and by which genes can be classified into three major categories, namely biological process, molecular function, and cellular component, and their sub-categories [34]. Out of the 41,483 most significant BLASTX hits against the NR plant species dataset, a total of 3,577 tea unigenes were assigned 14,283 GO term annotations using BLAST2GO [35]. These 14,283 GO terms were summarized into the three main GO categories and 43 sub-categories (Table 2 and Figure 5a). Cellular component made up the majority of the GO annotations (5,529, 38.7% of the total), followed by biological process (5,155, 36.1%) and molecular function (3,599, 25.2%). The major sub-categories are shown in Figure 5a: three sub-categories of "cell" (GO: 0005623), "cell part" (GO: 0044464) and "organelle" (GO: 0043226) were in the cluster of cellular component; two sub-categories of "binding functions" (GO: 0005488) and "catalytic functions" (GO: 0003824) were in the cluster of molecular function; and six sub-categories of "metabolic process" (GO: 0008152), "cellular process" (GO: 0009987), "biological regulation" (GO: 0065007), "establishment of localization" (GO: 0051234), "localization" (GO: 0051179), and "pigmentation" (GO: 00437473) were in the cluster of biological process. However, this result showed only a small proportion of tea unigenes with GO distributions, possibly due to large number of uninformative gene descriptions of these plant protein hits.

The 50,214 of BLASTX matches to *Arabidopsis *proteins was used for further GO mappings to improve the coverage and depth of GO annotation, because the *Arabidopsis *dataset has been extensively annotated with GO terms and the most abundant annotation records for *C. sinensis *unigenes were obtained from the comparisons with *Arabidopsis *proteins. As a result, the number of *C. sinensis *unigenes assigned with GO terms greatly increased to 32,107 (25% of the 127,094 unigenes). A total of 157,650 GO terms were associated with these 32,107 unigenes and classified into 41 functional sub-categories (Table 2 and Figure 5b). We discovered that the cluster of biological process was dominant (96,296, 61% of the total GO terms), in which the three sub-categories "response to stimulus" (GO: 0050896), "developmental process" (GO: 0032502), and "multicellular organismal process" (GO: 0032501) were included in the top 6 abundant sub-groups (in addition to the other three sub-categories of "cellular process," "metabolic process" and "biological regulation"). The major sub-categories in the clusters of cell component and molecular function were identical to the above mentioned GO classification. Moreover, only a few genes were assigned with "biological adhesion" (GO: 0022610), "locomotion" (GO: 0040011), "viral reproduction" (GO: 0016032) or "electron carrier" (GO: 0009055) GO terms. Out of 41 functional sub-categories, unigenes were sorted into the two previously unrepresented sub-categories of "rhythmic process" (GO: 0048511; 251 unigenes) and "viral reproduction" (GO: 0016032; 30 unigenes), based on *Arabidopsis *GO terms. No unigenes were annotated with "virion" (GO: 0019012), "virion part" (GO: 0044423), "chemoattractant" (GO: 0042056) or "chemorepellent" (GO: 0045499) GO terms. As a whole, 32,599 (26% of the 127,094 unigenes) of the unigenes were associated with at least one GO terms between the two kinds of GO annotations. These GO annotations demonstrate that *C. sinensis *expressed genes encoding diverse structural, regulatory and stress proteins.

#### KEGG Pathway mapping

In order to identify the biological pathways active in *C. sinensis*, the assembled unigenes were annotated with corresponding Enzyme commission (EC) numbers from BLASTX alignments against the KEGG database [36]. By mapping EC numbers to the reference canonical pathways, a total of 16,939 unigenes were assigned to 214 KEGG pathways (Table 2 and Figure 3a). The pathways most represented by unique sequences were inositol phosphate metabolism (1,348 members), benzoate degradation via CoA ligation (1,262 members), starch and sucrose metabolism (781 members) and purine metabolism (371 members).

Taken together, 53,966 unique sequence-based annotations had BLAST scores exceeding our threshold (≤1e^-5^) in NR, Uniprot and KEGG databases (Figure 3a). The Venn diagram (Figure 3c) shows that an additional 1,122 unigenes were annotated by domain-based alignments. Overall, 55,088 unique sequence-based or domain-based annotations using the six selected public databases were assigned to *C. sinensis *unigenes (43.3% of the assembled unigenes). Among them, 9,139 unigenes had hits in all six public databases with relatively defined functional annotations (Table 2). These annotations provide a valuable resource for investigating specific processes, structures, functions, and pathways in tea research.

### Analysis of metabolic pathway genes using *C. sinensis *unigenes

**Figure 6:**
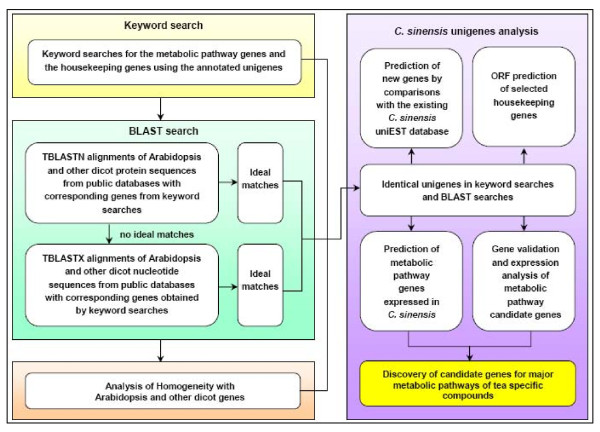
**Overall flow chart of the analysis of metabolic pathway genes using *C. sinensis *unigenes**.

The 55,088 annotated unigenes represented a significant expansion of the knowledge contained in existing *C. sinensis *EST libraries. Analysis of metabolic pathway genes using *C. sinensis *unigenes was accomplished as illustrated in Figure 6. We selected several primary and secondary metabolic pathways that are related to tea quality for further analysis. For functional annotations, we started with simple keyword searches and confirmed each search result with BLAST searches. These new genes and their annotations were compared with genes listed in the existing tea uniEST database [5-7].

#### Primary metabolic pathways in *C. sinensis*

The primary metabolic pathways selected included glycolysis (10 genes), citrate cycle (10 genes), pentose phosphate cycle (6 genes), and Calvin cycle and photosynthesis (12 genes). Our dataset includes annotated sequences for all genes in the selected four primary metabolic pathways, except for the glucose-6-phosphatase gene in the Calvin cycle (Additional File 3). Pyruvate kinase of the glycolysis pathway has the greatest number of singletons (23) matching the description of the gene. The rest of the primary metabolic genes have between 1 and 20 singletons matching each gene. The genes of the primary metabolic pathways displayed high homology to *Arabidopsis *or other dicot genes, with most of the genes having more than 80% similarity at the protein levels (data not shown), suggesting that these genes were highly conserved during the evolution.

#### Secondary metabolic pathways in *C. sinensis*

##### Flavonoid Biosynthesis

**Figure 7:**
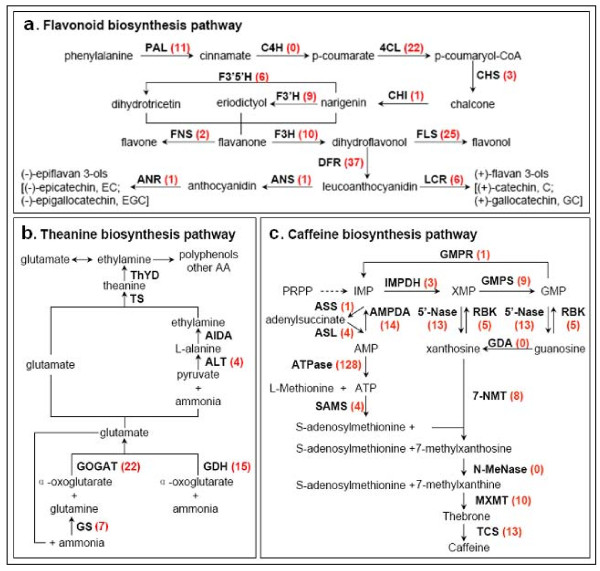
***C. sinensis *unigenes involved in three secondary metabolic pathways**. (a) *C. sinensis *unigenes involved in the pathway of flavonoid biosynthesis. (b) *C. sinensis *unigenes involved in the pathway of theanine biosynthesis. Putative theanine biosynthesis pathway is based on Sasaoka K (No. 48 in References). (c) *C. sinensis *unigenes involved in the pathway of caffeine biosynthesis. The red number in the bracket following each gene name indicates the number of corresponding *C. sinensis *unigenes.

Flavonoids are a class of important secondary metabolites including flavanones, flavones, dihydroflavonols, flavonols, and flavan 3-ols (catechins). They are important for tea quality and human health, especially catechins [37]. They also play a crucial role in plant defense against pathogens. In tea, flavonoids accumulate to higher levels in young leaves than in mature leaves [5]. In our annotated *C. sinensis *transcriptome dataset, multiple transcripts encoding almost all known enzymes involved in the main flavonoid biosynthesis pathway were identified (Figure 7a). Unigene IDs from the flavonoid biosynthesis pathway are listed in Additional File 4. Flavonoids are derived from phenylalanine and converted to chalcone via the phenylpropanoid pathway by the enzymes phenylalanine ammonia lyase (EC 4.3.1.24, 11 unigenes), cinnamate 4-hydroxylase (EC 1.14.13.11, no annotated unigenes available), 4-coumarate CoA ligase (EC 6.2.1.12, 22 unigenes), and chalcone synthase (EC 2.3.1.74, 3 unigenes). Subsequently, chalcone isomerase (EC 5.5.1.6, 1 unigene) catalyzes the stereo-specific cyclization of chalcones into naringenin (5, 7, 4'-trihydroxyflavanone). Naringenin can be converted by flavonoid 3'-hydroxylase (EC 1.14.13.21, 9 unigenes) and flavonoid 3', 5'-hydroxylase (EC 1.14.13.88, 6 unigenes) to produce eriodictyol (5,7,3',4'-tetrahydroxyflavanone) and dihydrotricetin (5,7,3',4',5'-pentahydroxyflavanone), respectively. Naringenin, eriodictyol and dihydrotricetin are the flavanones. Almost all flavonoid compounds can be derived from these flavanones. Flavone synthase (EC 1.14.11.2, 2 unigenes encoding FNSⅡ) catalyzes the conversion of flavanones to flavones, but flavanone 3-hydroxylase (EC 1.14.11.9, 10 unigenes) can convert these flavanones to dihydroflavonols. Following this reaction, the divergent conversions of dihydroflavonols include production of flavonols [catalyzed by flavonol synthase (EC 1.14.11.23, 25 unigenes)] and flavan-3,4-diols (leucoanthocyanidin) [catalyzed by dihydroflavonol 4-reductase (EC 1.1.1.219, 37 unigenes)]. Leucoanthocyanidins are the direct precursors to (+)-flavan 3-ols (e.g. (+)-catechin, (+)-gallocatechin) produced by leucoanthocyanidin reductase (EC 1.17.1.3, 6 unigenes). Formation of (-)-epiflavan 3-ols (e.g. (-)-epicatechin, (-)-epigallocatechin) can be achieved by a two-step reaction on leucoanthocyanidin by anthocyanidin synthase (EC 1.14.11.19, 1 unigenes) and anthocyanidin reductase (EC 1.3.1.77, 1 unigene). The above descriptions showed the vertical pathway responsible for the formation and conversion of the sub-categories of flavonoids. It is worth mentioning that flavonoid biosynthesis also includes multiple, parallel sub-pathways, which result from flavonoid 3'-hydroxylase and flavonoid 3', 5'-hydroxylase also accepting the flavones, dihydroflavonols, and flavonols as substrates (data not shown in Figure 7a). Therefore, flavonoid biosynthesis looks more like a complex metabolic grid [38] than a linear pathway. Since almost all the genes from the flavonoid pathway appeared to have more than one copy in the genome, we suspect *C. sinensis *encountered genome duplication events during the course of evolution.

Some genes of the phenylpropanoid pathway have been reported previously in *C. sinensis*. Some members have been expressed in *Escherichia coli *and were characterized [39-44] to be involved in catechin biosynthesis. We were able to detect these reported genes in our dataset (Additional File 4). Surprisingly, some genes associated with isoflavonoid metabolism were also discovered in our transcriptome, including isoflavone reductase (EC 1.3.1.-, 8 unigenes) and isoflavone 7-O-methyltransferase (EC 2.1.1.150, 1 unigene). The tea plant is not known to produce isoflavones. Similar discoveries have been reported in other non-isoflavone accumulating plants [45], suggesting homologs of isoflavone reductase and isoflavone O-methyltransferase may have general metabolic functions in many plant species.

In addition, new structural genes encoding flavonoid-modifying enzymes were discovered by a sequence or domain search on our dataset against the existing nucleotide and protein sequences of *C. sinensis *in NCBI or Uniprot databases. Most of these new genes are involved in glycosylation of flavonoids, catalyzing the transfer of either glucose from UDP-glucose or rhamnose from UDP-L-rhamnose to the hydroxyl groups of flavonoids (Additional File 4). For example, we found unigene sequences encoding tetrahydroxychalcone 2'-glucosyltransferase (1 unigene), flavonol-3-O-glycoside-7-O-glucosyltransferase 1 (6 unigenes), flavonol 3-O-glucosyltransferase/flavonoid-3-O-glucosyltransferase (EC 2.4.1.91, 7 unigenes), anthocyanidin 3-O-glucosyltransferase (EC 2.4.1.115, 33 unigenes), anthocyanidin 5,3-O-glucosyltransferase (EC 2.4.1.-, 12 unigenes), flavonol 3-O-glucoside L-rhamnosyltransferase (EC 2.4.1.159, 5 unigenes), and flavanone 7-O-glucoside 2''-O-beta-L-rhamnosyltransferase (EC 2.4.1.236, 10 unigenes). Moreover, unigenes encoding flavonol 3-O-methyltransferase (EC 2.1.1.76, associated with methylation of flavonols, 9 unigenes) and flavonol 3-sulfotransferase (EC 2.8.2.25, 2 unigenes) were also found in our annotated transcriptome. These genes represent an extensive repertoire of flavonoid modification enzymes that have not been reported before, although some of the conjugated compounds themselves have been identified.

Furthermore, a singleton for the flavonoid-related R2R3 transcription factor, a MYB4a repressor, was found. It is a member of the MYB transcription factor family that has been shown to interact with promoters of the phenylpropanoid pathway genes [46].

##### Theanine Biosynthesis

Theanine is a unique amino acid constituting 1-2% of the dry weight of tea leaf. It is synthesized in roots from glutamic acid and ethylamine by theanine synthetase (TS, EC 6.3.1.6; Figure 7b) [47,48]. The substrate ethylamine is derived from decarboxylation of alanine [49]. Newly synthesized theanine is translocated into the tender shoots through the xylem, where it either accumulates or is broken down into glutamic acid and ethylamine by theanine hydrolase (ThYD, EC 3.5.1.65) [50]. The enzymes involved in theanine synthesis also include glutamine synthetase (GS, EC 6.3.1.2), glutamate synthase (Fe-GOGAT, EC 1.4.7.1), glutamate dehydrogenase (GDH, EC 1.4.1.3), alanine transaminase (ALT, EC 2.6.1.2), and alanine decarboxylase (AIDA). Most of these theanine pathway genes were found in our dataset, except for *AIDA *and *ThYD*, which are specific to tea plants with no orthologs from other species be found in the public databases (Figure 7b; unigene IDs in the theanine biosynthesis pathway are listed in Additional File 4.). The *AIDA *sequences here were selected from the homologues of arginine decaboxylase (ADC) and S-adenosylmethionine decarboxylase (SAMDC), which have similar domains to *AIDA *[51]. The published *TS *was highly homologous to *GS*, thus the identified singletons needs to be confirmed enzymatically. Gamma-glutamyl transpeptidase (GGT, EC 2.3.2.2), used to synthesize theanine in bacteria [52-54], was chosen for a comparative study of the expression abundance in three different organs (see below).

##### Caffeine Biosynthesis

Caffeine (1, 3, 7-trimethylxanthine) is a purine alkaloid present in high concentrations in the tea plant. The caffeine biosynthesis pathway (Figure 7c) is part of the purine metabolism and is catalyzed by three S-adenosyl-L-methionine- (SAM) dependent N-methylation steps, namely xanthosine → 7-methylxanthosine → 7-methylxanthine → theobromine → caffeine. Different N-methyltransferases play important roles in the three N-methylation steps [55]. All related genes could be found in our transcriptome except the two genes guanosine deaminase (EC 3.5.4.15) and N-methylnucleosidase (EC 3.2.2.25) (Figure 7c; unigene IDs are listed in Additional File 4.). These two genes have not been cloned previously and thus do not exist in public databases.

#### ORF prediction of selected housekeeping gene

To further confirm our gene prediction and annotation algorithms, six housekeeping gene families were selected for open reading frame (ORF) analysis, namely actin, tubulin, histone, glyceraldehyde-3-phosphate dehydrogenase, 28S ribosomal protein, phosphofructokinase (Additional File 5). Out of a total of 88 unigene sequences, 62 sequences were predicted to contain the complete ORF, suggesting that among the house keeping genes, 70.5% of the genes already have the completed ORF. The deduced amino acid sequences have at least 68% homology to *Arabidopsis *or other dicot genes (data not shown).

#### Comparison of *C. sinensis* transcriptome with four *Camellia* cDNA libraries

Previously, we generated four cDNA libraries using different tissues of the *C. sinensis *plant. These tissues included young root, young leaf, subtractive young leaf, and drought-stressed root. The cDNAs were sequenced using the conventional Sanger sequencing. We compared these four cDNA libraries with our Illumina sequences using local BLASTN and TBLASTX.

**Figure 8:**
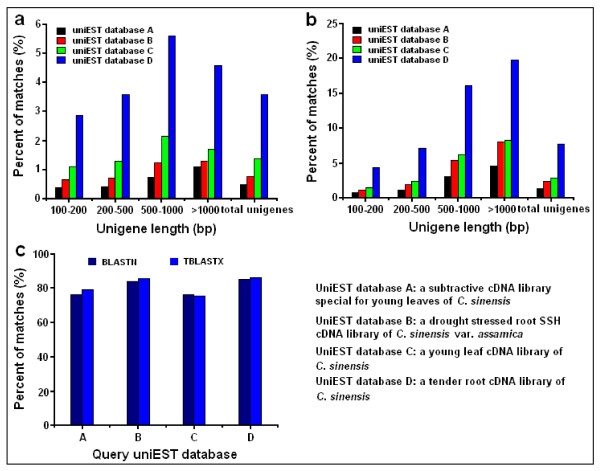
**BLAST comparisons of the *C. sinensis *transcriptome with four UniEST databases**. (a) Comparisons of the *C. sinensis *transcriptome to four uniEST databases using BLASTN algorithm. (b) Comparisons of *C. sinensis *transcriptome to four uniEST databases using TBLASTX algorithm. (c) Comparisons of four uniEST databases to *C. sinensis *transcriptome using both BLASTN and TBLASTX algorithms.

First, all EST sequences from the four selected *Camellia *cDNA libraries were assembled by Cap3 procedure [56] to obtain 1,809 uniESTs in the young root library, 921 uniESTs in the tender shoot library, 239 uniESTs in the young leaves subtractive cDNA library, and 419 uniESTs in the drought-stressed root SSH (suppression subtractive hybridization) library of *C. sinensis var. assamica*. We found that less than 5% of the unigenes from the Illumina transcriptome dataset have significant homology to the four uniEST databases at the nucleotide level, and less than 10% at the amino acid level. The alignment of *C. sinensis *transcriptome against the young root cDNA library produced the maximum number of best hits (Figure 8a, Figure 8b). These significant hits covered 75~85% of uniESTs in the four cDNA libraries (Figure 8c). In addition, unigenes in the *C. sinensis *transcriptome were further classified into four groups by sequence length, and each group was also aligned against the four uniEST databases. As expected, longer sequences produced more significant blast hits (Figure 8b). These results indicated that our transcriptome data have a high degree of consistency with previous EST data, yet also represent a significant increase in coverage.

#### Gene validation and expression analysis

**Figure 9:**
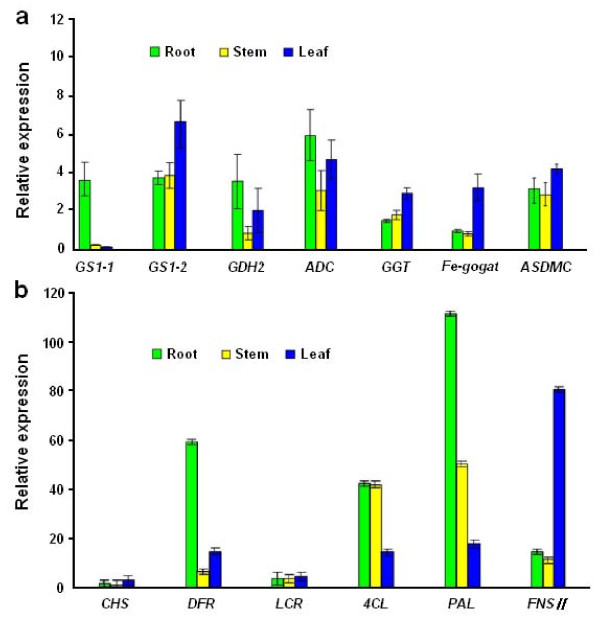
**Validation of candidate unigenes in *C. sinensis *transcriptome by qRT-PCR**. (a) Seven candidate unigenes involved in the theanine metabolic pathway show differential expression patterns by qRT-PCR in three organs. (b) Six candidate unigenes involved in the flavonoid biosynthesis show differential expression patterns by qRT-PCR in three organs. Results represent the mean (± SD) of three experiments.

To experimentally confirm that the unigenes obtained from sequencing and computational analysis were indeed expressed, 13 unigenes related to theanine and flavonoid synthesis were chosen for RT-PCR and qRT-PCR analysis (Figure 9a and Figure 9b).

In the RT-PCR analysis, every selected unigene was PCR positive with a single band at the calculated size (data not shown). In the qRT-PCR analysis, relative transcript levels of the unigenes from three different organs were further compared. In the theanine pathway, tea homologues of *GDH2*, *GS1-1 *and *ADC *were expressed much higher in young roots than in leaves and stems (Figure 9a), with the lowest expression level in the stems. In comparison, the expression levels of *GS1-2*, *GGT*, glutamate synthase (*Fe-GOGAT*) and *SAMDC *were low in young roots, and high in young shoots. The results confirmed that the expression of these selected genes directly correlated with theanine metabolism.

In the flavonoid pathway, six selected unigenes have differential expression patterns (Figure 9b). Overall expression levels of *PAL*, *4CL*, *DFR *and *FNSII *were higher than those of *CHS *and *LCR*. The tea homologues of *DFR *and *PAL *were highly expressed in young roots; *4CL *expression levels in roots were similar to those in stems. In contrast, the expression levels of *FNSII*, *CHS *and *LCR *in leaves were higher than those in roots. The results of qRT-PCR expression analysis matched the putative functions of these unigenes.

## Discussion

Ultra-high-throughput mRNA sequencing technology is a fast, efficient, and cost-effective way to characterize the poly (A)^+ ^transcriptome. It is especially suitable for gene expression profiling in non-model organisms that lack genomic sequences. To date, most sequencing efforts in *C. sinensis *were based on EST sequencing, with a limited number of tags reported in public databases. In this study, we applied RNA-seq technology for *C. sinensis *transcriptome profiling, in which the poly (A)^+ ^transcriptome was sequenced on the Illumina GA IIx platform. We obtained 2.59 G bp coverage with 34.5 million high-quality reads. We generated a total of 127,094 unigenes (≥100 bp) by *de novo *assembly. Among them, 55,088 assembled unigenes were annotated. Our coverage is approximately 10-fold more than all *C. sinensis *sequences deposited in GenBank combined (as of August 2010).

Since *C. sinensis *is self-incompatible and recalcitrant to genetic manipulations, little genetic or genomic information is currently available. Therefore, instead of a comprehensive in-depth investigation of the tea transcriptome, our experiment was designed to generate a quick landscape view. A number of strategies were adopted to obtain sufficient coverage of expressed transcripts, to improve the accuracy of *de novo *assembly, and to increase the effectiveness of the gene annotations. First, experimental materials for RNA preparation came from seven organs of the tea plant, which were selected to acquire as comprehensive coverage as possible. Second, an Illumina library was constructed based on the fragmenting RNA method, which has been shown both to reduce the amount of RNA secondary structure and 5' bias and to have better overall uniformity [11]. Third, a paired-end library sequencing strategy was applied not only to increase the sequencing depth, but also to improve the efficiency of *de novo *assembly. Finally, all six public databases were selected for gene annotation comparisons in order to acquire complete functional information.

As a result, 55,088 unigenes (43.3% of all assembled unigenes) returned significant hits from BLAST comparisons with the six public databases. These unigenes were assigned not only gene or protein name descriptions, but also putative conserved domains, gene ontology terms and metabolic pathways. Detailed functional information is important to understand overall expression profiles of *C. sinensis*. In particular, the number of unigenes that hit all six public databases summed up to 9,139. Because these genes had relatively unambiguous annotation, they were selected for tea-specific pathway analyses. The remaining 72,006 unigenes (56.7% of all assembled unigenes) did not generate significant homology to existing genes. The absence of homology could be caused by several factors. Obviously, a large proportion (82.1%) of unigenes was shorter than 500 bp, some of which were too short to allow statistically meaningful matches. However, for some unigenes, the absence of homologous sequences in the public databases may indicate specific roles for them in *C. sinensis*. We are currently cataloging the longer unigenes (≥500 bp; 22,757 unigenes) in tea plants.

The annotated unigenes were used to study primary and secondary metabolic pathways. For the four primary metabolic pathways investigated, all essential structural genes were found (Additional File 3). The putative pathway genes from tea were highly similar to those from the model dicot plant *Arabidopsis *or other dicot plants. We also analyzed six families of house-keeping genes to evaluate the completeness of our transcriptome coverage. More than 70.5% of these high copy number genes had full-length ORFs. We believe future large-scale sequencing efforts on tea genome and transcriptome will increase the coverage of our dataset even further.

The quality of tea in large part depends on its metabolic profiles. We focused on flavonoids, theanine and caffeine biosynthesis for additional analyses. We were able to find almost all metabolic genes from these pathways (Figure 7 and Additional File 4). Many of these genes appeared to form multi-gene families. It implies that the tea genome, like many other higher plants, went through one or more round of genome duplications during evolution. *C. sinensis *has a diploid genome, thus, extensive genome re-arrangement might have occurred. We are interested in using SNPs analysis to better understand the genome structure when more RNA-seq data has been obtained.

A few genes are currently missing in these pathways, which might be due to their low expression, insufficient sampling, or ineffective annotations. Some of these genes, such as guanosine deaminase and N-methylnucleosidase, have not been reported in plants before. On the other hand, we found some genes that might play important roles in the above mentioned pathways. For example, many glycosylation enzymes and cytochrome P450 genes were discovered in the transcriptome, which might contribute to the extensive modifications of various secondary compounds found in tea leaf extracts.

By comparing our transcriptome data with four previously prepared cDNA libraries analyzed by EST sequencing, we showed that the number of unigenes from RNA-seq was approximate 20 times more than the existing cDNA libraries. Yet, a small number of genes discovered in the cDNA libraries did not generate BLAST hits in the Illumina transcriptome, which could be resolved by increasing the sequencing depth, enhancing the accuracy of the assembly, and perfecting gene annotation strategies. We have selected two sets of structural enzymes to validate our gene annotations. Each one of them generated the expected band size by RT-PCR, and qRT-PCR analysis showed consistent expression patterns. We are confident that our transcriptome dataset is a valuable addition to the publicly available tea genomic information.

## Conclusions

Using Illumina sequencing technology, we surveyed the poly (A)^+ ^transcriptome of *C. sinensis *at an unprecedented depth (2.59 gigabase pairs) and produced 127,094 assembled unigenes with 55,088 unigenes obtaining annotation. To our knowledge, our results represent approximately 10-fold more genes than all *C. sinensis *genes deposited in GenBank (as of August 2010) and approximate 20 times more than the existing *C. sinensis *cDNA libraries. This study demonstrated that the Illumina sequencing technology could be applied as a rapid and cost-effective method for *de novo *transcriptome analysis of non-model plant organisms without prior genome annotation. These findings provided a comprehensive enough coverage to discover almost all known genes of several major metabolic pathways, which also serves as a substantial contribution to existing sequence resources for the tea plant. We believe that this transcriptome dataset will serve as an important public information platform to accelerate research of gene expression, genomics, and functional genomics in *C. sinensis*.

## Methods

### RNA preparation

Total RNA was extracted by modified CTAB method [57] from seven different tissues of the tea plant [*Camellia sinensis *(L.) O. Kuntze cv. *Longjing 43*], including tender shoots, young leaves, mature leaves, stems, young roots, flower buds and immature seeds which were snap-frozen and stored at -70°C until processing. RNA integrity was confirmed using the Agilent 2100 Bioanalyzer with a minimum integrity number value of 8. Equal amounts of total RNA from each tissue were pooled together for cDNA preparation.

### Preparation of cDNA library for transcriptome sequencing

The poly (A)^+ ^RNA was isolated from 20 μg of the total RNA pool using Dynal oligo(dT) 25 beads (Invitrogen) according to the manufacturer's protocol. Following purification, the mRNA was fragmented into smaller pieces at 70°C for 5 min in the fragmentation buffer (Ambion) and reverse-transcribed to synthesize first strand cDNA using SuperScript III reverse transcriptase (Invitrogen) and N6 random hexamers (Takara). Subsequently, second strand cDNA was synthesized using RNase H (Invitrogen) and DNA polymerase (Invitrogen). These cDNA fragments were further processed by an end repair using T4 DNA polymerase, Klenow DNA polymerase, and T4 polynucleotide kinase (NEB), and ligation of adaptors with Illumina's adaptor oligo mix and T4 DNA ligase (Invitrogen). The products were purified for the section of approximate 200 bp long using Qiaquick Gel Extraction Kit (Qiagen) and enriched with PCR for preparing the sequencing library. The cDNA library was detected by Agilent 2100 Bioanalyzer.

### Illumina sequencing

The cDNA library was sequenced from both of 5' and 3' ends on the Illumina GA IIx platform according to the manufacturer's instructions. The fluorescent images process to sequences, base-calling and quality value calculation were performed by the Illumina data processing pipeline (version 1.4), in which 75 bp paired-end reads were obtained.

### *De novo* assembly of sequencing reads and sequence clustering

Before assembly, the raw reads were filtered to obtain the high-quality clean reads by removing adaptor sequences, duplication sequences, the reads containing more than 10% "N" rate (the "N" character representing ambiguous bases in reads), and low-quality reads containing more than 50% bases with Q-value ≤ 5. The Q-value is the quality score assigned to each base by the Illumina's base-caller Bustard from the Illumina pipeline software suite (version 1.4), similar to the Phred score of the base call. *De novo *assembly of the clean read*s *was performed using SOAPdenovo program (version 1.03, http://soap.genomics.org.cn) which implements a de Bruijn graph algorithm and a stepwise strategy [28]. Briefly, the clean reads were firstly split into smaller pieces, the 'k-mers', for assembly to produce contigs using the de Bruijn graph. The resultant contigs would be further joined into scaffolds using the paired-end reads. Gap fillings were subsequently carried out to obtain the complete scaffolds using the paired-end information to retrieve read pairs that had one read well-aligned on the contigs and another read located in the gap region [58]. To reduce any sequence redundancy, the scaffolds were clustered using the Gene Indices Clustering Tools (copyright(c), http://compbio.dfci.harvard.edu/tgi/software/) [29]. The clustering output was passed to CAP3 assembler [56] for multiple alignment and consensus building. Others that can not reach the threshold set and fall into any assembly should remain as a list of singletons.

### Functional annotation and classification

All Illumina assembled unigenes (consensuses and singletons) longer than 200 bp were annotated by the assignments of putative gene descriptions, conserved domains, Gene Ontology terms, and putative metabolic pathways to them based on sequence similarity with previously identified genes annotated with those details. For assignments of predicted gene descriptions, the assembled unigenes were compared to the plant protein dataset of NR, the *Arabidopsis *protein dataset of NR, and Swiss-Prot/Uniprot protein database respectively using BLASTALL procedure (ftp://ftp.ncbi.nih.gov/blast/executables/release/2.2.18/) with a significant threshold of E-value ≤ 10^-5^. To parse the features of the best BLASTX hits from the alignments, putative gene names, 'CDS', and predicted proteins of corresponding assembled sequences can be produced. At the same time, the orientation of Illumina sequences which failed to be obtained directly from sequencing can be derived from BLAST annotations. For other sequences falling beyond the BLAST, ESTScan program (version 3.0.1, http://www.ch.embnet.org/software/ESTScan.html) was used to predict the 'CDS' and orientation of them. And then, since a large portion of assembled unigenes have not yet been annotated, conserved domains/families were further identified in the assembled unigenes using the InterPro database (version 30.0, HMMpfam, HMMsmart, HMMpanther, FPrintScan, ProfileScan, and BlastProDom) [30], Pfam database (version 24.0) [32] and Clusters of Orthologous Groups database at NCBI (as of December 2009, ftp://ftp.ncbi.nih.gov/pub/wolf/COGs/) [33]. Domain-based comparisons with the InterPro, Pfam and COGs databases were performed using InterProScan (version 4.5, ftp://ftp.ebi.ac.uk/pub/software/unix/iprscan/), HMMER3 (http://hmmer.janelia.org) and BLAST programs (E-value threshold: 10^-5^), respectively. Functional categorization by Gene Ontology terms (GO; http://www.geneontology.org) [34] was carried out based on two sets of best BLASTX hits from both the plant and *Arabidopsis *protein datasets of NR database using Blast2GO software (version 2.3.5, http://www.blast2go.de/) [35] with E-value threshold of 10^-5^. The KEGG pathways annotation was performed by sequence comparisons against the Kyoto Encyclopedia of Genes and Genomes database [36] using BLASTX algorithm (E-value threshold: 10^-5^).

Files containing the raw read sequences and their quality scores are available from the National Center for Biotechnology Information (NCBI) Short Read Archive with the accession number: SRX020193. The assembled sequences (longer than 150 bp) have been deposited in the Transcriptome Shotgun Assembly Sequence Database (TSA) at NCBI with the accession numbers: HP701085-HP777243.

### Analysis of metabolic pathway genes using *C. sinensis *unigenes

Genes involved in four primary pathways (glycolysis, citrate cycle, pentose phosphate cycle, and Calvin cycle and photosynthesis pathways) and three secondary metabolic pathways (flavonoid biosynthesis, theanine biosynthesis, and caffeine biosynthesis pathways) that are related to tea quality were analyzed using *C. sinensis *unigenes as illustrated in Figure 6. They were firstly searched based on standard gene names and synonyms in the combined functional annotations, and each search result was further confirmed with BLAST searches. BLAST searches were performed by the alignments of *Arabidopsis *or other dicot protein sequences from the public databases against corresponding genes obtained from keyword searches using the local TBLASTN alignments with an E-value threshold of 1e^-5^. If no hits can be produced from TBLASTN alignments, *Arabidopsis *or other dicot nucleotide sequences should be downloaded and used for additional TBLASTX alignments (E-value threshold: 1e^-5^). The identical results from keyword searches and BLAST searches can be used to predict that these genes could be expressed in *C. sinensis*. To discover new genes, all searched unigenes were analyzed by BLAST alignments against the existing tea uniEST database [5-7].

Six kinds of housekeeping genes were also searched by the combination method of simple text searches and BLAST searches. The ORFs of the selected housekeeping genes were predicted using the online ORF finder program (http://www.ncbi.nlm.nih.gov/gorf/gorf.html), and their homology to the model dicot plant *Arabidopsis *or other dicot plants were also analyzed.

### Comparisons with four *Camellia *cDNA libraries

The four *Camellia *cDNA libraries based on conventional Sanger sequencing were selected for the comparisons with *C. sinensis *transcriptome. The corresponding EST sequences from the four cDNA libraries were downloaded from the available *Camellia *ESTs in GenBank, including the EST sequences from the young root cDNA library of the tea plant (*C. sinensis*) submitted by our laboratory [7] (GenBank accession: GE652554.1-FE861258.1), two reported *C. sinensis *cDNA library respectively named subtractive cDNA library special for young leaves of the tea plant [5](GenBank accession: CV699876.1-CV699527.1) and the young leaf cDNA library of the tea plant [6](GenBank accession: CV067174.1-CV013548.1), and another drought-stressed root SSH cDNA library of *C. sinensis *var. *assamica *(GenBank accession:GW316945.1-GT969202.1). All EST sequences were assembled by Cap3 procedure [56] to obtain uniESTs with overlap length cutoff of 30nt and overlap percent identity parameter of 80. Comparisons of the *C. sinensis *transcriptome with the four *Camellia *cDNA libraries were performed with local BLASTN and TBLASTX procedures (1e^-5 ^cut-off), respectively.

### Gene validation and expression analysis

Thirteen selected unigenes with potential roles in theanine and flavonoid synthesis were chosen for validation using real time qPCR with gene specific primers designed with primer premier software (version 5.0) (Additional File 6). Total RNA was extracted from young roots, stems and young shoots of the tea plant using a modified CTAB method [57] and purified with RNA purification kit (Tiangen, China). One microgram of total RNA was used in reverse transcription in total volume of 20 μL in the presence of 6-mer random primer and oligo primer according to the protocol of Taraka. The standard curve for each gene was obtained by real-time PCR with several dilutions of cDNA. The reaction was performed in 20 μL, containing 10 μL 2×SYBR Green Mastermix (Taraka), 300 nM each primer and 2 μL 10-fold diluted cDNA template.

The PCR reactions were run in Bio-Rad Sequence Detection System using the following program: 95°C for 10 s and 40 cycles of 95°C for 15 s, anneal at 60°C for 30 s. Consequently, the specificity of the individual PCR amplification was checked using a heat dissociation protocol from 55 to 95°C following the final cycle of the PCR and agarose gel electrophoresis. Triplicates of each reaction were performed, and actin gene was chosen as an internal control for normalization after comparison the expressions of four reference genes (actin, GAPDH, 18S and β- tubulin) in different organs. Quantifying the relative expression of the genes in three different organs was performed using the delta-delta C_t _method as described by Livak and Schmittgen [59]. All data were expressed as the mean ± SD after normalization.

## Authors' contributions

XCW, TX, CJJ, ZZZ and CLW conceived and designed the experimental plan. CLW, HY, JS, YYL and QC participated in sample collection and RNA preparation. CYS and HY performed experiments. HY analyzed and interpreted the sequence data. CYS analyzed the qRT-PCR data. CYS and HY deposited the sequencing data in the GenBank database. HY, CYS, OY and XCW drafted and revised the manuscript. All authors read and approved the final manuscript.

## Supplementary Material

Additional file 1 **Top BLAST hits from the NCBI NR database**. BLAST results against the NCBI NR database for all the unigenes with E-value ≤ 1.0e^-5 ^are shown. Click here for file

Additional file 2 **List of Pfam domain families assigned to *C. sinensis *unigenes**. Domain-based search results from the Pfam database for all the unigenes with E-value ≤ 1.0e^-5 ^are shown. Click here for file

Additional file 3 **List of relative unigenes from four primary metabolic pathways in the *C. sinensis *transcriptome**. *C. sinensis *unigenes involved in four primary metabolic pathways, namely glycolysis, citrate cycle, pentose phosphate cycle, and Calvin cycle associated with photosynthesis, are listed. Click here for file

Additional file 4 **List of relative unigenes from three secondary metabolic pathways in the *C. sinensis *transcriptome**. *C. sinensis *unigenes involved in three secondary metabolic pathways, namely flavonoids biosynthesis, theanine biosynthesis, and caffeine biosynthesis, are listed. Click here for file

Additional file 5 **Complete ORF prediction analysis of housekeeping genes**. Six housekeeping gene families selected for ORF analysis include actin, tubulin, histone, glyceraldehyde-3-phosphate dehydrogenase, 28S ribosomal protein, and phosphor-fructokinase. Click here for file

Additional file 6 **Primers of the candidate unigenes designed for qRT-PCR**. Specific primers of thirteen candidate unigenes with potential roles in theanine and flavonoid biosynthesis designed for real time qRT-PCR using the Primer Premier program (version 5.0) are shown. Click here for file
