# Supplementary material for: Deep sequencing of the Camellia sinensis transcriptome revealed candidate genes for major metabolic pathways of tea-specific compounds
Source: BMC Genomics. 2011 Feb 28;12:131. doi: 10.1186/1471-2164-12-131 (PMC3056800; doi:10.1186/1471-2164-12-131)
Supplement: Additional file 3 — List of relative unigenes from four primary metabolic pathways in the C. sinensis transcriptome. C. sinensis unigenes involved in four primary metabolic pathways, namely glycolysis, citrate cycle, pentose phosphate cycle, and Calvin cycle associated with photosynthesis, are listed. [file 1471-2164-12-131-S3.DOC]

**List of relative unigenes from four primary metabolic pathways in the *C. sinensis* transcriptome**

| **Primary Metabolic pathway** | **Gene Name** | | | **No.** | **Unigene ID** |
| --- | --- | --- | --- | --- | --- |
| Glycolysis | HK | hexokinase [EC:2.7.1.1] | | 13 | Singletons112721, Singletons120289, Singletons124942, Singletons125996, Singletons16654, Singletons18467, Singletons21135, Singletons29171, Singletons34147, Singletons40325, Singletons50213, Singletons51630, Singletons52745 |
|  | GPI | Phosphoglucose isomerase /glucose-6-phosphate isomerase [EC:5.3.1.9] | | 9 | Singletons109244, Singletons116669, Singletons120561, Singletons121780, Singletons124990, Singletons20665, Singletons25416, Singletons36534, Singletons978 |
|  | PFK | 6-phosphofructokinase [EC:2.7.1.11] | | 17 | Singletons121389, Singletons122437, Singletons122787, Singletons124018, Singletons25579, Singletons2961, Singletons38390, Singletons42162, Singletons43741, Singletons44431, Singletons49223, Singletons4964, Singletons50099, Singletons50374, Singletons53318, Singletons53669, Singletons6699 |
|  | ALDO | fructose-bisphosphate aldolase [EC:4.1.2.13] | | 6 | Singletons123636, Singletons18986, Singletons36670, Singletons41045, Singletons41640, Singletons6842 |
|  | TPI | triosephosphate isomerase [EC:5.3.1.1] | | 2 | Singletons32620, Singletons38419 |
|  | GAPDH | glyceraldehyde-3-phosphate dehydrogenase [EC:1.2.1.12] | | 8 | Singletons108192, Singletons12150, Singletons22771, Singletons36049, Singletons37527, Singletons40009, Singletons42106, Singletons49631 |
|  | PGK | phosphoglycerate kinase [EC:2.7.2.3] | | 4 | Singletons25618, Singletons27329, Singletons32724, Singletons7667 |
| Glycolysis | PGM | phosphoglycerate mutase [EC:5.4.2.1] | | 20 | Singletons107735, Singletons115066, Singletons124909, Singletons125846, Singletons125890, Singletons28681, Singletons28877, Singletons30387, Singletons3499, Singletons37512, Singletons38963, Singletons42527, Singletons46663, Singletons47172, Singletons47344, Singletons49676, Singletons52170, Singletons52439, Singletons54185, Singletons9628 |
|  | ENO | enolase [EC:4.2.1.11] | | 4 | Singletons17921, Singletons23107, Singletons46136, Singletons6373 |
|  | PK | Pyruvate kinase [EC:2.7.1.40] | | 23 | Cluster1235_Consensus1, Singletons120256, Singletons124070, Singletons125008, Singletons125905, Singletons12859, Singletons13337, Singletons20042, Singletons20941, Singletons27244, Singletons2800, Singletons29025, Singletons30987, Singletons32524, Singletons36864, Singletons40386, Singletons44321, Singletons45098, Singletons47015, Singletons51402, Singletons53261, Singletons803, Singletons9336 |
| Citrate cycle | CS | citrate synthase [EC:2.3.3.1] | | 4 | Singletons117346, Singletons123406, Singletons35117, Singletons54810 |
|  | ACO | aconitase [EC:4.2.1.3] | | 17 | Singletons109932, Singletons115935, Singletons123454, Singletons124765, Singletons125157, Singletons1532, Singletons21909, Singletons22754, Singletons28567, Singletons36329, Singletons38528, Singletons39311, Singletons42940, Singletons43399, Singletons43564, Singletons5192, Singletons8464 |
|  | IDH | isocitrate dehydrogenase (NAD+) [EC:1.1.1.41] | | 11 | Singletons109713, Singletons111744, Singletons120056, Singletons120539, Singletons121053, Singletons17822, Singletons31684, Singletons36661, Singletons39791, Singletons52458, Singletons54343 |
| Citrate cycle | α-KGDH | alpha- ketoglutarate dehydrogenase complex | alpha-ketoglutarate dehydrogenase /2-oxoglutarate dehydrogenase [EC:1.2.4.2] | 5 | Singletons112123, Singletons124502, Singletons25864, Singletons44947, Singletons54365 |
|  |  |  | dihydrolipoyl succinyltransferase [EC:2.3.1.61] | 7 | Singletons10193, Singletons10417, Singletons116393, Singletons123030, Singletons28231, Singletons33018, Singletons33055 |
|  |  |  | dihydrolipoyl dehydrogenase [EC:1.8.1.4] | 5 | Singletons108859, Singletons115155, Singletons120029, Singletons29250, Singletons30512 |
|  | SUCLG | succinyl-CoA synthetase /succinyl-CoA ligase (GDP-forming) [EC:6.2.1.4] | | 7 | Singletons113309, Singletons119395, Singletons1919, Singletons33639, Singletons41628, Singletons45109, Singletons48076 |
|  | SDH | succinate dehydrogenase [EC:1.3.5.1] | | 3 | Singletons110473, Singletons120546, Singletons39951 |
|  | FUM | fumaras/fumarate hydratase [EC:4.2.1.2] | | 3 | Singletons121110, Singletons125847, Singletons49219 |
|  | MDH | malate dehydrogenase [EC:1.3.1.37] | | 10 | Singletons124123, Singletons13688, Singletons15218, Singletons30409, Singletons31984, Singletons34603, Singletons36294, Singletons41767, Singletons46527, Singletons48793 |
| Pentose phosphate cycle | G6PD | glucose-6-phosphate dehydrogenase /glucose-6-phosphate 1-dehydrogenase [EC:1.1.1.49] | | 19 | Cluster1207_Consensus1, Cluster1656_Consensus1, Singletons110267, Singletons110294, Singletons116909, Singletons124304, Singletons125972, Singletons15678, Singletons18175, Singletons20678, Singletons26342, Singletons30345, Singletons33745, Singletons37246, Singletons38374, Singletons38406, Singletons45340, Singletons4859, Singletons8562 |
|  | PGLS | 6-phosphogluconolactonase [EC:3.1.1.31] | | 4 | Singletons12628, Singletons18058, Singletons37521, Singletons53135, |
| Pentose phosphate cycle | PGD | 6-phosphogluconate dehydrogenase [EC:1.1.1.44] | | 6 | Singletons117096, Singletons14178, Singletons1877, Singletons22793, Singletons30106, Singletons8990 |
|  | RPE | ribulose-5-phosphate 3-epimerase /pentose-5-phosphate 3-epimerase [EC:5.1.3.1] | | 3 | Singletons123371, Singletons24966, Singletons50058 |
|  | TKTL | transketolase [EC:2.2.1.1] | | 6 | Singletons22046, Singletons23111, Singletons3467, Singletons53133, Singletons53845, Singletons7252 |
|  | TALDO | transaldolase [EC:2.2.1.2] | | 6 | Singletons108011, Singletons108292, Singletons121361, Singletons29663, Singletons46297, Singletons53185 |
| Clavin cycle | RuBisCO | ribulose 1,5--biophosphate carboxylase/oxygenase [EC:4.1.1.39] | | 5 | Singletons115702, Singletons15010, Singletons16895, Singletons2959, Singletons7718 |
|  | PGK | phosphoglycerate kinase [EC:2.7.2.3] | | 4 | Singletons25618, Singletons27329, Singletons32724, Singletons7667 |
|  | GAPDH | glyceraldehyde-3-phosphate dehydrogenase [EC:1.2.1.12] | | 8 | Singletons108192, Singletons12150, Singletons22771, Singletons36049, Singletons37527, Singletons40009, Singletons42106, Singletons49631 |
|  | TIM | triosephosphate isomerase [EC:5.3.1.1] | | 2 | Singletons32620, Singletons38419 |
|  | TALDO | transaldolase [EC:2.2.1.2] | | 6 | Singletons108011, Singletons108292, Singletons121361, Singletons29663, Singletons46297, Singletons53185 |
|  | FBPase | fructose-1,6-bisphosphatase [EC:3.1.3.11] | | 6 | Singletons120068, Singletons16087, Singletons20379, Singletons35488, Singletons49090, Singletons53942 |
|  | GPI | glucose-6-phosphate isomerase /glucosephosphate isomerase [EC:5.3.1.9] | | 9 | Singletons109244, Singletons116669, Singletons120561, Singletons121780, Singletons124990, Singletons20665, Singletons25416, Singletons36534, Singletons978 |
|  | G6Pase | glucose-6-phosphatase [EC:3.1.3.9] | | 0 | --* |
| Clavin cycle | TKTL | transketolase [EC:2.2.1.1] | | 6 | Singletons22046, Singletons23111, Singletons3467, Singletons53133, Singletons53845, Singletons7252 |
|  | SBPase | sedoheptulose 1,7-bisphosphatase [EC:3.1.3.37] | | 2 | Singletons12593, Singletons23144 |
|  | RPI | phosphopentose isomerase /ribose-5-phosphate isomerase [EC:5.3.1.6] | | 1 | Singletons5903 |
|  | PRK | phosphoribulokinase /ribulose-5-phosphate kinase [EC:2.7.1.19] | | 2 | Singletons481, Singletons4886 |

--*: No unigene sequences currently were available.
