# Supplementary material for: Deep sequencing of the Camellia sinensis transcriptome revealed candidate genes for major metabolic pathways of tea-specific compounds
Source: BMC Genomics. 2011 Feb 28;12:131. doi: 10.1186/1471-2164-12-131 (PMC3056800; doi:10.1186/1471-2164-12-131)
Supplement: Additional file 4 — List of relative unigenes from three secondary metabolic pathways in the C. sinensis transcriptome. C. sinensis unigenes involved in three secondary metabolic pathways, namely flavonoids biosynthesis, theanine biosynthesis, and caffeine biosynthesis, are listed. [file 1471-2164-12-131-S4.DOC]

**List of relative unigenes from three secondary metabolic pathways in the *C. sinensis* transcriptome**

| **Secondary Metabolic Pathway** | **Gene Name** | | **No.** | **Unigene ID** |
| --- | --- | --- | --- | --- |
| Flavonoid Biosynthesis | PAL | phenylalanine ammonia lyase [EC:4.3.1.24] | 11 | Singletons10462, Singletons109363, Singletons122892, Singletons18657, Singletons23476, Singletons26104, Singletons27358, Singletons44643, Singletons46825, Singletons6222, Singletons8728 |
|  | C4H | cinnamate 4-hydroxylase [EC:1.14.13.11] | 0 | --* |
|  | 4CL | 4-coumarate CoA ligase [EC:6.2.1.12] | 22 | Cluster704_Consensus1, Singletons108886, Singletons109022, Singletons11041, Singletons114080, Singletons11643, Singletons120702, Singletons121814, Singletons125641, Singletons19458, Singletons22452, Singletons33310, Singletons36489, Singletons37183, Singletons41539, Singletons42786, Singletons47605, Singletons49493, Singletons4995, Singletons53107, Singletons582, Singletons8618 |
|  | CHS | chalcone synthase [EC:2.3.1.74] | 3 | Singletons125814, Singletons983, Singletons9897 |
|  | CHI | chalcone isomerase [EC:5.5.1.6] | 1 | Singletons20391 |
|  | F3’H | flavonoid 3’-hydroxylase [EC:1.14.13.21] | 9 | Singletons114493, Singletons126059, Singletons28032, Singletons2830, Singletons29857, Singletons36439, Singletons43380, Singletons45148, Singletons48548 |
|  | F3’5’H | flavonoid 3’,5’-hydroxylase [EC:1.14.13.88] | 6 | Singletons115745, Singletons123966, Singletons126059, Singletons2830, Singletons29857, Singletons45148 |
| Flavonoid Biosynthesis | FNSII | flavone synthase II [EC:1.14.11.22] | 2 | Singletons123323, Singletons45946 |
|  | F3H | flavanone 3-hydroxylase [EC:1.14.11.9] | 10 | Singletons10115, Singletons11471, Singletons120051, Singletons121009, Singletons16915, Singletons32537, Singletons34332, Singletons5502, Singletons5614, Singletons9923 |
|  | FLS | flavonol synthase [EC:1.14.11.23] | 25 | Singletons114429, Singletons116776, Singletons120028, Singletons122048, Singletons125447, Singletons21623, Singletons24187, Singletons25536, Singletons27350, Singletons31273, Singletons34750, Singletons41460, Singletons41923, Singletons41976, Singletons42884, Singletons42995, Singletons44287, Singletons44364, Singletons44808, Singletons45333, Singletons46817, Singletons49925, Singletons53367, Singletons6568, Singletons9522 |
|  | DFR | dihydroxyflavonol 4-reductase [EC:1.1.1.219] | 37 | Singletons108243, Singletons10902, Singletons110657, Singletons111745, Singletons112402, Singletons113438, Singletons11699, Singletons123483, Singletons19257, Singletons19797, Singletons22157, Singletons23374, Singletons24418, Singletons25605, Singletons30867, Singletons31253, Singletons3367, Singletons3744, Singletons37730, Singletons42427, Singletons42591, Singletons42895, Singletons46392, Singletons46416, Singletons47660, Singletons48316, Singletons48361, Singletons50095, Singletons50343, Singletons51450, Singletons52515, Singletons54761, Singletons5770, Singletons5802, Singletons6598, Singletons6877, Singletons8836 |
|  | LCR | leucoanthocyanidin reductase [EC:1.17.1.3] | 6 | Singletons110657, Singletons111317, Singletons11699, Singletons121045, Singletons18389, Singletons51136 |
| Flavonoid Biosynthesis | ANS | anthocyanidin synthase [EC:1.14.11.19] | 1 | Singletons14705 |
|  | ANR | anthocyanidin reductase [EC:1.3.1.77] | 1 | Singletons18389 |
|  | THC2'GT | tetrahydroxychalcone 2'-glucosyltransferase [EC:2.4.1.-] | 1 | Singletons44580 |
|  | F3OG-7-OGT | flavonol 3-O-glycoside 7-O-glucosyltransferase 1 [EC:2.4.1.-] | 6 | Singletons123124, Singletons13346, Singletons17205, Singletons2223, Singletons26432, Singletons8237 |
|  | F3GT | flavonol 3-O-glucosyltransferase /flavonoid-3-O-glucosyltransferase [EC:2.4.1.91] | 7 | Singletons18492, Singletons29565, Singletons310, Singletons53958, Singletons120746, Singletons4410, Singletons9736 |
|  | 3-AOGT | anthocyanidin 3-O-glucosyltransferase [EC:2.4.1.115] | 33 | Singletons10130, Singletons108440, Singletons10928, Singletons110548, Singletons119229, Singletons119287, Singletons120618, Singletons120746, Singletons123956, Singletons15188, Singletons18492, Singletons23399, Singletons25708, Singletons25861, Singletons26266, Singletons32730, Singletons33025, Singletons38055, Singletons38574, Singletons42306, Singletons4535, Singletons45539, Singletons45697, Singletons4692, Singletons4792, Singletons49228, Singletons4990, Singletons5283, Singletons53284, Singletons5352, Singletons6350, Singletons654, Singletons9736 |
|  | 5,3-AOGT | anthocyanidin 5,3-O-glucosyltransferase [EC:2.4.1.-] | 12 | Singletons11717, Singletons14851, Singletons22068, Singletons2421, Singletons25165, Singletons28204, Singletons30615, Singletons36896, Singletons39285, Singletons40090, Singletons50251,Singletons7717 |
|  | F3OG-LRT | flavonol 3-O-glucoside L-rhamnosyltransferase [EC:2.4.1.159] | 5 | Singletons16998, Singletons28390, Singletons35170, Singletons41079, Singletons47520 |
| Flavonoid Biosynthesis | F7G | flavanone 7-O-glucoside 2''-O-beta-L-rhamnosyltransferase [EC:2.4.1.236] | 10 | Singletons124006, Singletons16573, Singletons17626, Singletons24070, Singletons27744, Singletons29267, Singletons3012, Singletons4606, Singletons5700, Singletons715 |
|  | 3-FOMT | flavonol 3-O-methyltransferase [EC:2.1.1.76] | 9 | Singletons118737, Singletons119268, Singletons123216, Singletons1238, Singletons2967, Singletons41434, Singletons44177, Singletons54196, Singletons54538 |
|  | F3ST | flavonol 3-sulfotransferase [EC:2.8.2.25] | 2 | Singletons126067, Singletons370 |
|  | F4'ST | flavonol 4'-sulfotransferase [EC:2.8.2.27] | 15 | Singletons108304, Singletons109438, Singletons114024, Singletons116679, Singletons125142, Singletons18, Singletons24212, Singletons26170, Singletons36898, Singletons370, Singletons39777, Singletons40357, Singletons44368, Singletons49638, Singletons8659 |
|  | FSTL | Flavonol sulfotransferase-like | 10 | Singletons110313, Singletons110728, Singletons124976, Singletons20514, Singletons2779, Singletons28576, Singletons43093, Singletons49892, Singletons50022, Singletons6926 |
|  | IFR | isoflavone reductase [EC:1.3.1.-] | 8 | Singletons113967, Singletons121045, Singletons122277, Singletons42871, Singletons45124, Singletons47781, Singletons51136, Singletons54775 |
|  | 7-IOMT | isoflavone 7-O-methyltransferase [EC:2.1.1.150] | 1 | Singletons44177 |
| Theanine Biosynthesis | GS/ TS | glutamine synthetase [EC:6.3.1.2]/ Theanine synthetase [EC:6.3.1.6] | 7 | Singletons11049 , Singletons117214, Singletons28501, Singletons36347, Singletons 64941, Singletons79559, Singletons8405 |
| Theanine Biosynthesis | GOGAT- NADPH  /NADH | glutamate synthase (NADPH/NADH) [EC:1.4.1.13 1.4.1.14] | 14 | Singletons114365, Singletons13850, Singletons15174, Singletons18394, Singletons20994, Singletons21702, Singletons242, Singletons24769, Singletons2894, Singletons34171, Singletons35621, Singletons41025, Singletons41655, Singletons41676 |
|  | GOGAT-Fe | glutamate synthase (ferredoxin) [EC:1.4.7.1] | 8 | Singletons115174, Singletons115583, Singletons124797, Singletons126089, Singletons126094, Singletons17587, Singletons51262, Singletons53013 |
|  | GDH | glutamate dehydrogenase [EC:1.4.1.3] | 15 | Cluster882_Consensus1, Singletons110126, Singletons121757, Singletons122633, Singletons13609, Singletons17087, Singletons19692, Singletons25894, Singletons26015, Singletons35129, Singletons38078, Singletons39876, Singletons40369, Singletons41629, Singletons49662 |
|  | GGT | gamma-glutamyl transpeptidase [EC:2.3.2.2] | 5 | Singletons118124, Singletons120830, Singletons123496, Singletons125982, Singletons47906 |
|  | ALT | alanine aminotransferase [EC:2.6.1.2] | 4 | Singletons119816, Singletons46772, Singletons54113, Singletons6428 |
|  | ADC | arginine decarboxylase  [EC:4.1.1.19] | 3 | Singletons27593, Singletons4048, Singletons7913 |
|  | SAMDC | S-adenosylmethionine decarboxylase  [EC:4.1.1.50] | 6 | Singletons10394, Singletons114228, Singletons119089, Singletons15166, Singletons31796, Singletons35281 |
| Caffeine Biosynthesis | IMPDH | IMP dehydrogenase [EC:1.1.1.205] | 3 | Singletons118921, Singletons16616, Singletons31305 |
|  | GMPS | GMP synthase [EC:6.3.4.1] | 9 | Singletons126052, Singletons12676, Singletons14458, Singletons18625, Singletons21671, Singletons21983, Singletons27798,Singletons28629, Singletons35586 |
|  | GMPR | GMP reductase [EC:1.7.1.7] | 1 | Singletons44998 |
| Caffeine Biosynthesis | 5’-Nase | 5’-nucleotidase [EC:3.1.3.5] | 13 | Singletons110327, Singletons110409, Singletons123693, Singletons124513, Singletons126049, Singletons20755, Singletons24079, Singletons2707, Singletons27451, Singletons33043, Singletons34501, Singletons4280, Singletons47311 |
|  | RBK | ribokinase [EC:2.7.1.15] | 5 | Singletons119077, Singletons124272, Singletons21063, Singletons40261, Singletons53034 |
|  | GDA | guanosine deaminase [EC:3.5.4.15] | 0 | --* |
|  | ASS | adenylosuccinate synthase [EC:6.3.4.4] | 1 | Singletons32140 |
|  | ASL | adenylosuccinate lyase [EC:4.3.2.2] | 4 | Singletons117649, Singletons18397, Singletons31733, Singletons8435 |
|  | AMPDA | AMP deaminase [EC:3.5.4.6] | 14 | Singletons110160, Singletons117235, Singletons117884, Singletons23913, Singletons26709, Singletons27359, Singletons28767, Singletons28897, Singletons33340, Singletons36692, Singletons40333, Singletons41735, Singletons676, Singletons754 |
|  | SAMS | S-adenosylmethionine synthase [EC:2.5.1.6] | 4 | Singletons116600, Singletons1716, Singletons38366, Singletons53098 |
|  | 7-NMT | 7-methylxanthosine synthase [EC:2.1.1.158] | 8 | Singletons120444, Singletons124786, Singletons13634, Singletons18036, Singletons42084, Singletons53179, Singletons53574, Singletons54286 |
|  | N-MeNase | N-methylnucleotidase [EC:3.2.2.25] | 0 | --* |
|  | MXMT | theobromine synthase [EC:2.1.1.159] | 10 | Singletons114156, Singletons120444, Singletons124786, Singletons13634, Singletons18036, Singletons42084, Singletons53179, Singletons53574, Singletons54286, Singletons92818 |
| Caffeine Biosynthesis | TCS | Tea caffeine synthase [EC:2.1.1.160] | 13 | Singletons114156, Singletons120444, Singletons124786, Singletons13634, Singletons18036, Singletons42084, Singletons53179, Singletons53574, Singletons54286, Singletons92818, Singletons46822, Singletons47474, Singletons74979 |
|  | ATPase | ATPase [EC:3.6.-] | 128 | Cluster1473_Consensus1, Cluster1563_Consensus1, Cluster449_Consensus1, Cluster595_Consensus1, Cluster8_Consensus1, Singletons10424, Singletons10708, Singletons107800, Singletons107915, Singletons107967, Singletons108724, Singletons108875, Singletons10931, Singletons109571, Singletons10978, Singletons109846, Singletons110080, Singletons11081, Singletons111592, Singletons112405, Singletons113623, Singletons11464, Singletons114718, Singletons115585, Singletons115807, Singletons11596, Singletons115987, Singletons116530, Singletons116556, Singletons11776, Singletons11789, Singletons11793, Singletons118333, Singletons118799, Singletons118866, singletons118923, Singletons119327, Singletons119644, Singletons119770, Singletons120457, Singletons12062, Singletons121048, Singletons121368, Singletons121644,  Singletons122259, Singletons122357, Singletons122481, Singletons12278, Singletons122815, Singletons122940, Singletons123667, Singletons123984, Singletons124154, Singletons1243, Singletons124653, Singletons124929, Singletons125481, Singletons126151, Singletons126227, Singletons126288, Singletons12670, Singletons12937, Singletons13520, Singletons13726,  Singletons13988, Singletons14492, Singletons14617, Singletons14622, |
| Caffeine Biosynthesis | ATPase | ATPase [EC:3.6.-] | 128 | Singletons14708, Singletons15022, Singletons15165, Singletons15278, Singletons15799, Singletons15888, Singletons15941, Singletons16648, Singletons16983, Singletons16987, Singletons1781, Singletons18351, Singletons18494, Singletons18798, Singletons19109, Singletons2001,  Singletons20050, Singletons20295, Singletons20674, Singletons20733, Singletons21012, Singletons2110, Singletons21639, Singletons21721, Singletons21786, Singletons21815, Singletons21836, Singletons22241, Singletons22439, Singletons22692, Singletons23643, Singletons25178, Singletons25620, Singletons26085, Singletons2637, Singletons26414, Singletons265, Singletons26874, Singletons27123, Singletons27360, Singletons27411, Singletons27461, Singletons27734, Singletons27878, Singletons2799, Singletons28088, Singletons28186, Singletons2844, Singletons29125, Singletons2934, Singletons29727, Singletons29794, Singletons29970, Singletons30249, Singletons30530, Singletons31258, Singletons31277, Singletons31419, Singletons31716, Singletons31791 |

--*: No unigene sequences currently were available.
