# Supplementary material for: Deep sequencing of the Camellia sinensis transcriptome revealed candidate genes for major metabolic pathways of tea-specific compounds
Source: BMC Genomics. 2011 Feb 28;12:131. doi: 10.1186/1471-2164-12-131 (PMC3056800; doi:10.1186/1471-2164-12-131)
Supplement: Additional file 5 — Complete ORF prediction analysis of housekeeping genes. Six housekeeping gene families selected for ORF analysis include actin, tubulin, histone, glyceraldehyde-3-phosphate dehydrogenase, 28S ribosomal protein, and phosphor-fructokinase. [file 1471-2164-12-131-S5.DOC]

**Complete OFR prediction analysis of housekeeping genes**

| **Housekeeping genes** | **No.** | **No of genes with complete OFR** |
| --- | --- | --- |
| actin | 30 | 23 |
| tubulin | 27 | 15 |
| histone | 6 | 6 |
| glyceraldehyde-3-phosphate dehydrogenase | 8 | 5 |
| 28S ribosomal protein | 2 | 2 |
| phosphofructokinase | 15 | 11 |
| Total | 88 | 62 |
