# Supplementary material for: Deep sequencing of the Camellia sinensis transcriptome revealed candidate genes for major metabolic pathways of tea-specific compounds
Source: BMC Genomics. 2011 Feb 28;12:131. doi: 10.1186/1471-2164-12-131 (PMC3056800; doi:10.1186/1471-2164-12-131)
Supplement: Additional file 6 — Primers of the candidate unigenes designed for qRT-PCR. Specific primers of thirteen candidate unigenes with potential roles in theanine and flavonoid biosynthesis designed for real time qRT-PCR using the Primer Premier program (version 5.0) are shown. [file 1471-2164-12-131-S6.DOC]

**Primers of the candidate unigenes designed for** **qRT-PCR**

| **Gene ID** | **Putative function** | **Primer** | **Length of PCR products (bp)** |
| --- | --- | --- | --- |
| Singletons8099 | Actin | F: 5’-GCCATATTTGATTGGAATGG-3’  R: 5’-GGTGCCACAACCTTGATCTT-3’ | 207 |
| Singletons11049 | Glutamine synthetase1-1 (GS1-1) | F: 5’-TGGCTCTGGACTCCACTCTT-3’  R:5’-GCAGCCTTGAAGCATATTG-3’ | 209 |
| Singletons36347 | Glutamine synthetase1-2 (GS1-2) | F: 5’-CACAGAGAAAGCCGGAAAAG-3’  R:5’-TCAAATCCCCAACCAAACAT-3’ | 156 |
| Singletons41629 | Glutamate dehydrogenase2 (GDH2) | F: 5’-GGCGTGGTGTTATTTATGC-3’  R:5’-AATCCCGTTTGGGTTCTTA-3’ | 188 |
| Singletons4048 | Arginine decarboxylase (ADC) | F: 5’-GGGGATGTGCAGAGTGATTT-3’  R: 5’-GTGCTGGTTTCACCACCTTT-3’ | 175 |
| Singletons47906 | Gamma-glutamyl transpeptidase (GGT) | F: 5’-GACCACTTTGAAGCTCCTGC-3’  R:5’-GGATTCCTTAAAACCACCA-3’ | 210 |
| Singletons126089 | Glutamate synthase (Fe-GOGAT) | F: 5’-AGAATACGGAAGTAAAGAAACG-3’  R: 5’-TCACTGGAGTAGCCATAAGC-3’ | 160 |
| Singletons35281 | S-adenosylmethionine decarboxylase (ASDMC) | F: 5’-CGGTAATGATCTTGGTTCC-3’  R:5’-TTCTCGTCATCCTCGTTCT-3’ | 184 |
| Singletons125814 | Chalcone synthase (CHS) | F:5’-GTGCTCTGGCGGATTATGGA-3’  R:5’-CCCCACTCATCGCTGTTTTC-3’ | 118 |
| Singletons30867 | Dihydroxyflavonol 4-reductase (DFR) | F:5’- AGGGGCTTCAGGGTTCATAG-3’  R:5’-CTGCTTTCCACAGGGTTAGG-3’ | 158 |
| Singletons51136 | Leucoanthocyanidin reductase (LCR) | F:5’-ACATTTGCTGTAACTCCATCG-3’  R:5’-GACTGAGCCATCACCGTAG-3’ | 101 |
| Cluster704_Consensus1 | 4-coumarate CoA ligase (4CL) | F:5’-GAGACAATCAAACAAGCAA-3’  R:5’-TAAACACCCATCTGGAGGA-3’ | 140 |
| Singletons18657 | Phenylalanine ammonia-lyase (PAL) | F: 5’-GTCCAAAGCACCGAGCAAC-3’  R:5’-GTCCAAAGCACCGAGCAAC-3’ | 123 |
| Singletons45946 | Flavone synthase Ⅱ (FNSⅡ) | F: 5’-GCTGCCCTGGTACTTCTTTG-3’  R: 5’-CCATCACCGTTCATCACATC-3’ | 115 |
